# Chronic, Combined Cardiac and Renal Dysfunction Exacerbates Renal Venous Pressure-Induced Suppression of Renal Function in Rats

**DOI:** 10.3389/fphys.2022.781504

**Published:** 2022-02-04

**Authors:** Shereen M. Hamza, Xiaohua Huang, Tayyaba Zehra, Wenqing Zhuang, William A. Cupples, Branko Braam

**Affiliations:** ^1^Division of Nephrology, Department of Medicine, University of Alberta, Edmonton, AB, Canada; ^2^Department of Physiology, University of Alberta, Edmonton, AB, Canada; ^3^Biomedical Physiology and Kinesiology, Simon Fraser University, Burnaby, BC, Canada

**Keywords:** combined cardiac and renal dysfunction, renal venous congestion, renal blood flow, glomerular filtration rate, blood pressure

## Abstract

**Background and Objective:**

Increased renal venous pressure (RVP) is common in combined heart and kidney failure. We previously showed that acute RVP elevation depresses renal blood flow (RBF), glomerular filtration rate (GFR), and induces renal vasoconstriction in the absence of changes in blood pressure in healthy rats. We used our established rodent model of chronic combined heart and kidney failure (H/KF) to test whether RVP elevation would impair cardiovascular stability, renal perfusion and exacerbate renal dysfunction.

**Methods:**

Male rats were subjected to 5/6 nephrectomy (SN_x_ or Sham) and 6% high salt diet followed 7 weeks later by ligation of the left anterior descending coronary artery (CL or Sham). Experimental groups: CL + SN_x_ (*n* = 12), Sham CL + SN_x_ (*n* = 9), CL+ Sham SN_x_ (*n* = 6), and Sham Control (*n* = 6). Six weeks later, anesthetized rats were subjected to an acute experiment whereupon mean arterial pressure (MAP), heart rate (HR), RVP, RBF, and GFR were measured at baseline and during elevation of RVP to 20–25 mmHg for 120 min.

**Results:**

Baseline MAP, HR, RBF, and renal vascular conductance (RVC) were comparable among groups. Baseline GFR was significantly depressed in CL + SN_x_ and Sham CL + SN_x_ groups compared to Sham Control and CL + Sham SN_x_ groups. Upon RVP increase, MAP and HR fell in all groups. Increased RVP exacerbated the reduction in RBF in CL + SN_x_ (−6.4 ± 0.9 ml/min) compared to Sham Control (−3.7 ± 0.9 ml/min, *p* < 0.05) with intermediate responses in Sham CL + SN_x_ (−6.8 ± 1.3 ml/min) and CL + Sham SN_x_ (−5.1 ± 0.4 ml/min) groups. RVP increase virtually eliminated GFR in CL + SN_x_ (−99 ± 1%), Sham CL + SN_x_ (−95 ± 5%), and CL + Sham SN_x_ (−100%) groups compared to Sham Control (−84 ± 15% from baseline; *p* < 0.05). Renal vascular conductance dropped significantly upon RVP increase in rats with HF (CL + SN_x_: −0.035 ± 0.011; CL + Sham SN_x_: −0.050 ± 0.005 ml/min·mmHg^−1^, *p* < 0.05) but not Sham CL + SN_x_ (−0.001 ± 0.019 ml/min·mmHg^−1^) or Control (−0.033 ± mL/min·mmHg^−1^).

**Conclusion:**

Chronic combined heart and kidney failure primarily impairs renal hemodynamic stability in response to elevated RVP compared to healthy rats.

## Introduction

Significant evidence supports pathophysiological interactions between the heart and the kidneys. For example, chronic kidney disease is directly associated with increased risk of cardiac disease (Go et al., 2004). Similarly, cardiac dysfunction complicated by decreased kidney function is associated with adverse outcomes and poor prognosis (Damman et al., 2007; Bagshaw et al., 2013). As such, combined heart and kidney failure (H/KF), also referred to as the cardiorenal syndrome, describes a clinical condition in which dysfunction of one of these organ systems can initiate and amplify the dysfunction of the other (Bongartz et al., 2005; Ronco et al., 2008). Clinically, patients with this condition are exceedingly difficult to manage and morbidity and mortality is high as a result (Damman et al., 2007).

A major challenge in this field is an incomplete understanding of the nature of interactions between the heart and kidneys. This led our group to establish and characterize a rodent model of combined heart and kidney dysfunction (Bongartz et al., 2012a,b) which recapitulates key aspects of human cardiorenal syndrome such as decreased ejection fraction and a low glomerular filtration rate (GFR). An aspect of the model we have not investigated, and which has not been investigated by others, is renal venous pressure (RVP). Our previous studies have demonstrated that acute elevation of RVP impairs kidney function by reducing renal blood flow (RBF), renal vascular conductance (RVC), and GFR (Huang et al., 2018). Interestingly, we also demonstrated that an isolated and chronic increase in RVP in otherwise healthy rats initiates both anatomic and physiologic adaptations which serve to return RVP to the normal range (Hamza et al., 2020). These adaptations attenuate the renal functional response to a superimposed increase in RVP; however, this occurs at the cost of impaired ability to maintain stable blood pressure and reduced baseline renal perfusion (Hamza et al., 2020).

The overarching aim of this study was to clarify the role of RVP in this model of H/KF. We hypothesized that RVP elevation in this model of H/KF would impair cardiovascular stability, renal perfusion, and exacerbate renal dysfunction. To address this, we elevated RVP in our rodent model of cardiorenal syndrome and then studied the renal and systemic response.

## Materials and Methods

### Animals and Ethics

Male Lewis rats (250–300 g, 10–11 weeks of age, *n* = 33; Charles River, St. Constant, QC, Canada) were housed in a temperature and humidity-controlled room featuring a 12-h:12-h light/dark cycle within the University of Alberta animal facility for at least 1 week prior to surgical preparation. All rats were fed standard 0.3% sodium rodent chow and water *ad libitum*. All experiments were approved by the University of Alberta Animal Care and Use Committee in accordance with the guidelines issued by the Canada Council on Animal Care.

### Recovery Surgical Preparation to Induce Subtotal 5/6 Nephrectomy (SN_X_)

The protocol used for this study was similar to the protocol we used before, with slight modifications (Bongartz et al., 2012b). Briefly, rats were anesthetized with isoflurane; buprenorphine (0.02 mg/kg) and meloxicam (2 mg/kg) were administered s.c. Ophthalmic ointment (OptixCare, CLC MEDICA, Ontario, Canada) was applied to the rat’s eyes, and the right flank was shaved and skin cleansed with 3X—alternating applications of 10% povidone-iodine solution (Prepodyne, West Penetone Inc., Canada) and 70% ethanol. Under aseptic conditions, the right kidney was accessed *via* flank incision and the renal neurovascular bundle and right ureter were isolated and ligated with sterile 6-0 silk sutures (Braintree Scientific; Braintree, MA, United States). The right neurovascular bundle and ureter were then severed with surgical scissors, and the right kidney was completely removed, taking care to leave the right adrenal gland intact. Abdominal muscle and skin were then sutured closed in layers (3-0 Chromic Gut, Ethicon, NJ, United States) and the incision cleaned and treated with antiseptic ointment. The rats were then monitored and permitted to recover for 1 week. Surgical procedures were identical for sham-operated animals with the exception of ligation of the neurovascular bundle, ureter, and removal of the right kidney; renal structures were manipulated with sterile cotton swabs, and the neurovascular bundle and ureter were isolated by blunt dissection.

Following 1-week recovery, rats were again anesthetized and the left flank was prepared as described above. The left kidney was accessed *via* a flank incision and connective tissue attachments were removed from the kidney surface, taking care to leave the left adrenal gland intact. The rostral pole of the kidney (~1/3) was surgically resected and sterile gelatin foam sponge (Surgi-Foam, Johnson & Johnson Ethicon; United States) was applied to the cut surface with gentle pressure to induce hemostasis. Shortly thereafter, the caudal pole of the kidney (~1/3) was resected and gelatin foam sponge applied to induce hemostasis. Once the remnant kidney was no longer bleeding and perfusion was verified, the muscle and skin were closed in layers and the animal was permitted to recover as described above. Sham-operated animals were similarly prepared with the exception of surgical resection of kidney tissue. Ten days following this procedure, rats were switched from normal chow to 6% NaCl to induce volume expansion characteristic of this model. Rats then remained on this diet for the duration of the study.

### Recovery Surgical Procedure to Induce Myocardial Infarction and Cardiac Dysfunction

**Figure 1:**
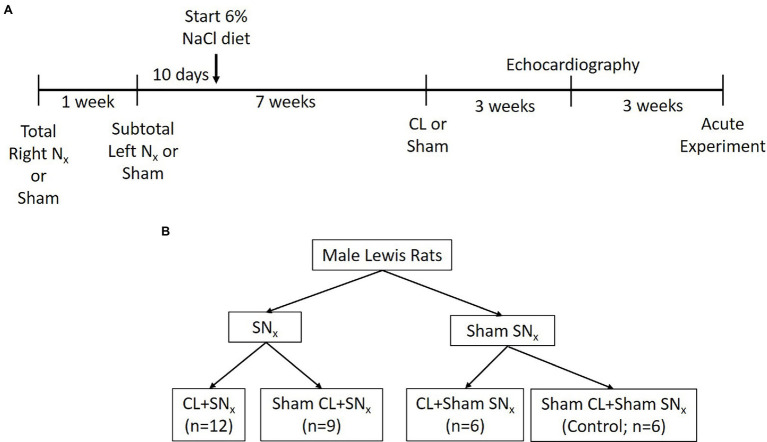
Schematic representation of surgical timelines **(A)** and experimental groups **(B)**.

Eight weeks following the initial SN_x_ or Sham operation, rats were then subjected to left anterior descending coronary artery ligation to induce myocardial infarction or sham operation as we have previously described (Bongartz et al., 2012b). Briefly, rats were anesthetized as described above, intubated, and subjected to mechanical ventilation. Lidocaine (10 mg/kg) was administered s.c. at this time to help prevent fatal arrythmia. The left side of the chest was shaved, and skin was cleansed as described above. An incision was made between the 4th and 5th intercostal muscles and blunt dissection exposed the heart. A suture (5-0 Ethilon, Johnson & Johnson) was tied around the left anterior descending coronary artery which induced a characteristic “blanching” of cardiac tissue downstream of the ligature. The chest was closed first (3-0 silk) followed by closure of overlying muscle and skin in layers (4-0 Vicryl). Isoflurane anesthesia was gradually reduced, and animals were removed from the ventilator before they regained consciousness. Sham-operated rats underwent the same procedure with the exception of tightening of the ligature around the coronary artery. Rats then recovered and were monitored for the duration of the study. Four to Five weeks after the coronary artery ligation procedure, a subset of rats from each experimental group (described below) were anesthetized and subjected to echocardiography to assess ejection fraction and verify the presence or absence of cardiac dysfunction (Figure 1A; Supplementary Table S1).

### Experimental Groups

The procedures described above resulted in four experimental groups as follows: combined heart and kidney dysfunction (CL + SN_x_; *n* = 12); Sham a (*n* = 9); CL + Sham SNx (*n* = 6); and Sham Control (*n* = 6; Figure 1B).

### Surgical Preparation for Acute Elevation of RVP

Preparation for the acute elevation in RVP was completed as we have previously described (Huang et al., 2018). Briefly, 6 weeks following recovery from coronary artery ligation or sham operation, rats were administered buprenorphine (0.01–0.02 mg/kg, S.C.) and subsequently anesthetized (0.5–4% isoflurane, 17 ml/min O_2_). Rats were then transferred to a heated surgical stage and body temperature maintained at 36.5–37°C (Vestavia Scientific, Birmingham, AL, United States). Ophthalmic ointment was applied to the rat’s eyes (OptixCare, CLC MEDICA, Ontario, Canada), and hair was manually shaved from the neck, abdomen, and left groin; skin was cleansed with 11% povidone-iodine solution. Tracheotomy was performed (PE 240, VWR, Alberta, Canada), and the tracheotomy tube was inserted into a rodent anesthesia nosecone adapted for this purpose. The femoral vein was cannulated (Silastic, VWR), and 5% bovine serum albumin +250 μg/min FITC Inulin in 0.9% NaCl was administered i.v. at 1.5 ml/h. The femoral artery was cannulated (PE 50, VWR) to permit direct recording of MAP and HR. Following midline laparotomy, the adrenal or supra-spermatic vein was cannulated (MRE-025, Braintree Scientific, Braintree, MA, United States) to permit direct recording of RVP. A 3-0 Prolene snare was placed around the renal vein at its junction with the inferior vena cava. The left ureter was cannulated (PE 10, VWR) for urine collection, and the renal artery was dissected free from the renal vein; a transit-time ultrasound probe (1RB series, Transonic Systems, Ithaca, NY, United States) was placed around the main trunk of the renal artery and covered in acoustic coupling gel (SurgiLube, Transonic Systems, Ithaca, NY, United States) for direct recording of RBF. The i.v. infusion solution was switched to 1% bovine serum albumin+250 μg/min FITC Inulin in 0.9% NaCl at 1.5 ml/h for maintenance.

### Experimental Protocol

Following a 35–45-min equilibration period, a baseline arterial blood sample (400 μl) was taken and baseline recording commenced for 1 h with urine collected every 30 min. At this time, rats were randomized to an RVP increase group in which the Prolene snare was partially occluded to superimpose further increase of RVP to 20–25 mmHg (RVP). Recording continued for a further 2 h, during which timed urine samples were collected every 30 min and arterial blood samples (300 μl) were collected every hour. At experiment end, rats were euthanized with sodium pentobarbital (80 mg/kg).

### Determination of GFR Using FITC Inulin

Collected plasma and urine samples were diluted in 0.5 mol/l HEPES (pH 7.4). 50 μl of each sample was loaded in duplicate onto a 96-well black plate (Greiner, Monroe, NC, United States). Fluorescence was determined using the Fluoroskan Ascent® Microplate Fluorometer (Thermo Fisher Scientific, Vantaa, Finland), at an excitation wavelength of 485 nm and emission wavelength of 527 nm.

### Data Analysis and Statistics

Data were collected and stored on a PC using a PowerLab Data Acquisition System (8/30; ADInstruments, Dunedin, New Zealand) and LabChart 6 Software. One-way ANOVA or two-way RM ANOVA with a Student–Newman–Keuls *post-hoc* test was used to evaluate baseline characteristics and the impact of elevated RVP as appropriate. Differences between groups were analyzed by three-way ANOVA with a Student–Newman–Keuls *post-hoc* test. Statistical significance was accepted at *p* < 0.05. All data are presented as means ± SEM.

## Results

### Combined Heart/Kidney Dysfunction Did Not Alter Baseline Cardiovascular Parameters but Reduced GFR

Baseline parameters between the four experimental groups were comparable at the time of the acute experiment (Supplementary Table S1). There were no statistically significant differences in baseline MAP and HR, although MAP in Sham CL + SN_x_ rats tended to be higher than the other groups. Baseline RVC was similar across all four experimental groups; however, baseline GFR was comparably and significantly reduced in the groups with reduced kidney mass (CL + SN_x_ and Sham CL + SN_x_) compared to those with intact kidney mass (CL + Sham SN_x_ and Sham Control, Supplementary Table S1, one-way ANOVA, *p* < 0.05). Baseline RVP was also comparable among experimental groups. Similarly, hematocrit at the time of the acute experiment was not different between the experimental groups and control, which was maintained for the duration of the protocol (Supplementary Table S2).

### Acute Elevation of RVP Induces an Abrupt Reduction in MAP Which Is Exacerbated by Heart or Kidney Failure Compared to Controls

**Figure 2:**
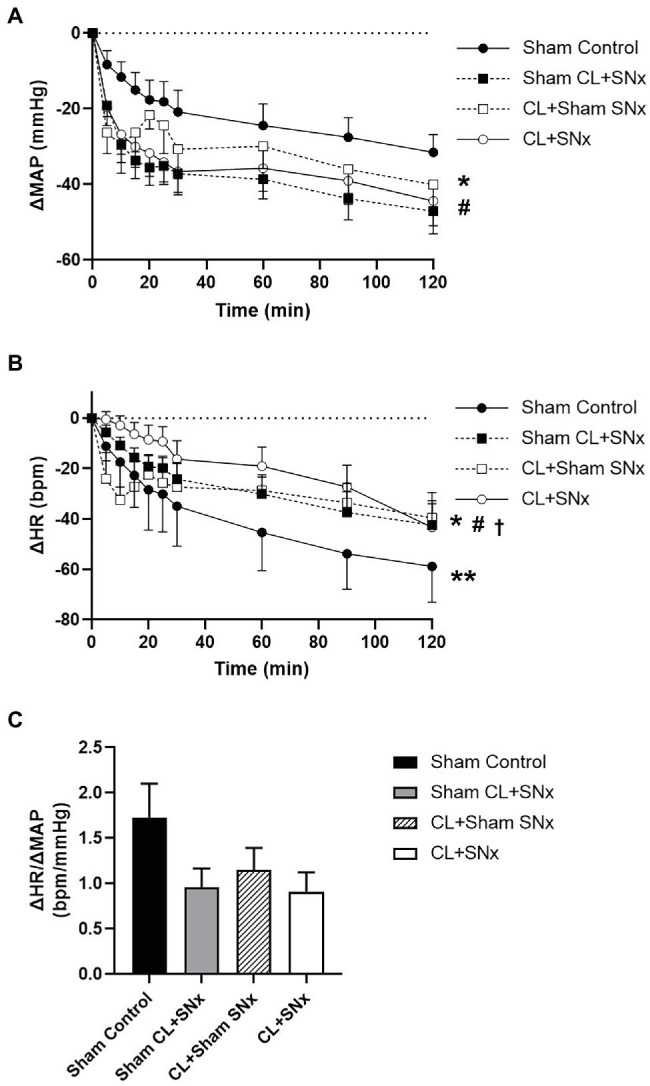
RVP increase induced a significant reduction in MAP and HR in all groups compared to baseline. **(A)** RVP elevation induced a significant change in MAP from baseline in all groups: CL + SN_x_ (*n* = 12), CL + Sham SN_x_ (*n* = 9), Sham CL + SN_x_ (*n* = 6), and Control (*n* = 6); one-way RM ANOVA, ^*^p < 0.05 compared to baseline within each group. MAP in Sham CL + SN_x_ and CL + Sham SN_x_ groups fell significantly lower than Controls (three-way ANOVA: SN_x_ vs. Sham SN_x_ within Sham CL, ^#^*p* < 0.05; CL vs. Sham CL within Sham SN_x_, ^#^*p* < 0.05) and were not significantly different from CL + SN_x_. **(B)** Acute RVP elevation induced a progressive reduction in HR from baseline within each group (one-way RM ANOVA, ^*^p < 0.05). HR fell to a greater degree in Control group compared to Sham CL + SN_x_ (three-way ANOVA: SN_x_ vs. Sham SN_x_ within Sham CL, ^**^p < 0.05) while HR fell to a greater degree in both Sham CL + SN_x_ and CL + Sham SN_x_ groups compared to CL + SN_x_ (three-way ANOVA: CL or Sham CL within SN_x_, ^#^*p* < 0.05); three-way ANOVA CL or Sham CL within Sham SN_x_, ^†^*p* < 0.05. **(C)** The ratio between ΔHR/ΔMAP was comparable between all four groups.

Acute elevation of RVP induced a progressive and significant reduction in MAP over time compared to baseline in all groups (one-way RM ANOVA, *p* < 0.05); MAP in Sham CL + SN_x_ and CL + Sham SN_x_ groups fell significantly lower than Controls (three-way ANOVA: SN_x_ vs. Sham SN_x_ within Sham CL, *p* < 0.05; CL vs. Sham CL within Sham SN_x_, *p* < 0.05) and were not significantly different from CL + SN_x_ (Figure 2A). The fall in MAP did not reach statistical significance between CL + SN_x_ and Sham Control groups. Acute RVP elevation induced a progressive reduction in HR from baseline in each group (one-way RM ANOVA, *p* < 0.05); HR fell to a greater degree in Control group compared to Sham CL + SN_x_ (three-way ANOVA: SN_x_ vs. Sham SN_x_ within Sham CL, *p* < 0.05) while HR fell to a greater degree in both Sham CL + SN_x_ and CL + Sham SN_x_ groups compared to CL + SN_x_ (three-way ANOVA: CL or Sham CL within SN_x_, *p* < 0.05; three-way ANOVA CL or Sham CL within Sham SN_x_, *p* < 0.05, Figure 2B). The RVP-induced fall in HR between CL + SN_x_ and Sham Control groups did not reach statistical significance. The ratio of the change in HR to the change in MAP (ΔHR/ΔMAP) at the end of the experimental recording period did not differ among groups (Figure 2C).

### H/KF Exacerbates the RVP-Induced Fall in RBF but Heart Failure Exacerbates RVC Depression

**Figure 3:**
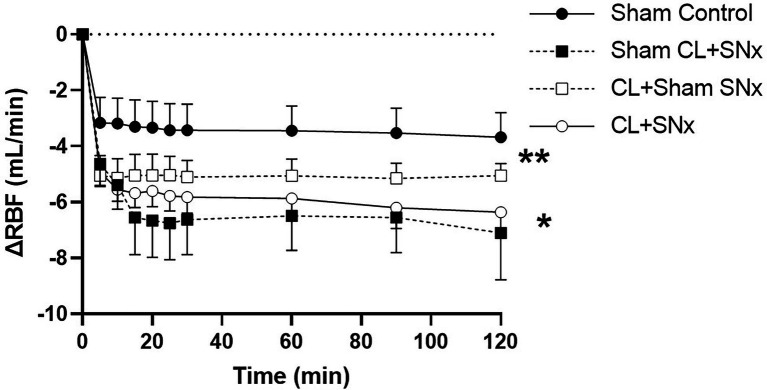
Acute RVP elevation induces a significant fall in RBF, which is exacerbated by combined heart/kidney failure. RVP elevation induced a significant fall in RBF from baseline over time within each group: CL + SN_x_ (*n* = 12), CL + Sham SN_x_ (*n* = 9), Sham CL + SN_x_ (*n* = 6), and Control (*n* = 6); one-way RM ANOVA, ^*^p < 0.05. The fall in RBF was significantly greater in the CL + SN_x_ group compared to Sham Control (two-way RM ANOVA, ^**^p < 0.05). The RVP-induced reduction in RBF in both CL + Sham SN_x_ and Sham CL + SN_x_ was not significantly lower than Control. The fall in RBF in CL + Sham SN_x_ and Sham CL+ SN_x_ was not significantly different from each other or CL + SN_x_.

**Figure 4:**
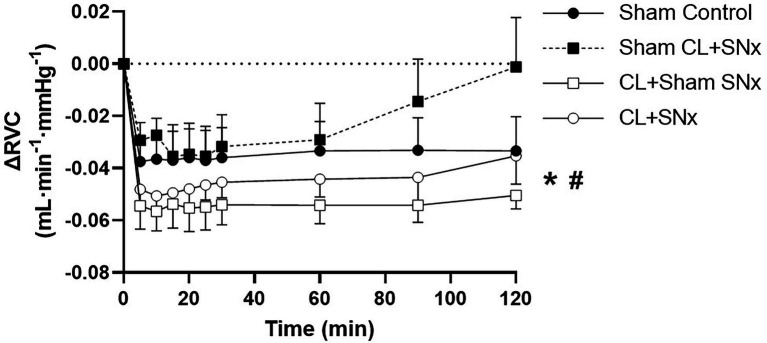
Heart failure exacerbated the RVP-induced fall in renal vascular conductance. Acute RVP elevation induced a significant fall in RVC from baseline in CL + SN_x_ (*n* = 12) and CL + Sham SN_x_ (*n* = 9) groups (one-way RM ANOVA, baseline vs. timepoints, ^*^p < 0.05); there was no statistically significant fall in RVC in Sham CL + SN_x_ (*n* = 6) or Sham Control (*n* = 6) groups. The magnitude of the change in RVC was greater in the groups subjected to coronary artery ligation (CL or Sham CL, three-way ANOVA, ^#^*p* < 0.05). The magnitude of RVC decrease was comparable between CL + Sham SN_x_ and CL + SN_x_ (SN_x_ or Sham SN_x_ within CL, three-way ANOVA).

Acute RVP elevation induced a significant fall in RBF from baseline over time in all groups (one-way RM ANOVA, *p* < 0.05, Figure 3). This reduction in RBF was significantly greater in the CL + SN_x_ group compared to Sham Control (two-way RM ANOVA, *p* < 0.05, Figure 3). The RVP-induced reduction in RBF in both CL+Sham SN_x_ and Sham CL + SN_x_ was not significantly lower than Control. The fall in RBF in CL + Sham SN_x_ and Sham CL + SN_x_ was not significantly different from each other or CL + SN_x_. In contrast, acute RVP elevation induced a significant fall in RVC from baseline in CL + SN_x_ and CL + Sham SN_x_ groups (one-way RM ANOVA, baseline vs. timepoints, *p* < 0.05, Figure 4); there was no statistically significant fall in RVC in Sham CL + SN_x_ or Sham Control groups. The magnitude of the change in RVC was greater in the groups subjected to coronary artery ligation (CL or Sham CL, three-way ANOVA, *p* < 0.05). The magnitude of RVC decrease was comparable between CL + Sham SN_x_ and CL + SN_x_ (SN_x_ or Sham SN_x_ within CL, three-way ANOVA; Figure 4).

### GFR Is Maintained in Only Sham Controls in Response to Acute RVP Elevation

**Figure 5:**
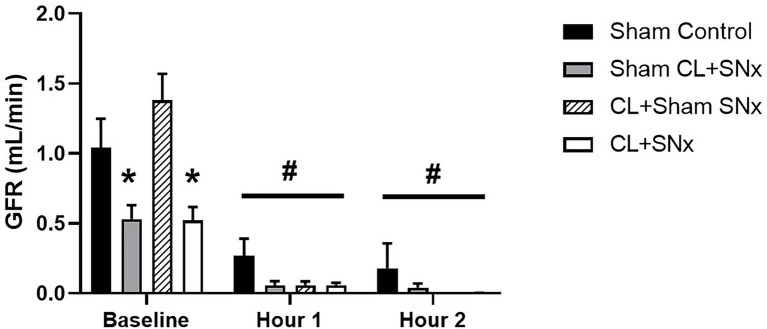
Acute RVP elevation induced a significant reduction of GFR in all experimental groups. Baseline GFR was significantly lower in Sham CL + SN_x_ and CL + SNx groups compared to Control and CL + Sham SN_x_; one-way ANOVA, ^*^p < 0.05. Upon RVP elevation, GFR fell significantly from baseline within all groups; one-way RM ANOVA compared to baseline, ^#^*p* < 0.05.

Although acute RVP elevation significantly reduced GFR within all groups compared to baseline (one-way RM ANOVA compared to baseline, *p* < 0.05), by the end of the experimental recording period, urine flow was virtually eliminated in CL + SN_x_, Sham CL + SN_x_, and CL + Sham SN_x_ groups, resulting in near-complete reduction of GFR (CL + SN_x_: −99.3 ± 0.6%; Sham CL + SN_x_: −95.4 ± 4% and CL + Sham SN_x_: −100%, Figure 5). In contrast, urine flow was reduced, but maintained in the Sham Control group such that GFR fell by −84 ± 14% from baseline. Due to the inherent variability of these measurements, as well as the technical difficulty in quantifying GFR introduced by the near cessation of urine flow, the magnitude of change in GFR in response to RVP elevation did not reach statistical significance between Sham Control and CL + SN_x_, Sham CL + SN_x_, and CL + Sham SN_x_ groups.

## Discussion

A compelling interaction exists between the heart and kidneys such that dysfunction in one organ can initiate and/or exacerbate dysfunction in the other. The precise nature of this interaction remains unclear; however, “cardiorenal connectors” have been recognized (Bongartz et al., 2005; Yogasundaram et al., 2019). In addition, hemodynamic factors such as venous congestion, specifically increased RVP, appear to figure prominently. We have previously demonstrated that both acute and chronic increase in RVP impacts renal and systemic hemodynamics in healthy rats (Huang et al., 2018; Hamza et al., 2020). In the current study, which is the first of its kind, we extend these findings with an investigation of the impact of elevated RVP in a rodent model of combined H/KF that we previously developed and characterized (Bongartz et al., 2012b).

We demonstrate that elevated RVP significantly depresses blood pressure in all groups. Compared to controls, this fall in blood pressure is more pronounced in animals with either heart or kidney failure; the blood pressure response in these two groups was similar to rats with combined H/KF in the current study, but direct comparison between controls and rats with combined H/KF did not reach statistical significance. Increased RVP also induced a significantly greater fall in RBF in the context of combined H/KF. This exaggerated fall in RBF is due, in part, to renal vasoconstriction because the fall in RVC was also greater in animals with heart failure alone or combined H/KF than in controls and those with renal failure alone. The pronounced fall in RBF may not be completely related to the RVP-induced fall in MAP as RBF fell to a greater degree than would be anticipated based on a reduction in renal perfusion pressure alone. It is plausible that renal venous congestion induced an increase in renal interstitial pressure (Winton, 1931; Fiksen-Olsen et al., 1992; Komuro et al., 2018) with consequent compression of the intrarenal vasculature and tubules that was enhanced in the remnant kidney in our study. Based on hemodynamic principles, the ensuing increase in peritubular capillary pressure represents an impediment to RBF. Furthermore, the increase in tubular intraluminal pressure would reduce glomerular transcapillary hydrostatic pressure gradients, effecting a significant reduction in GFR (Leyssac et al., 1991; Ross, 2012). GFR, albeit at a low level, was maintained only in the Sham controls in response to RVP elevation and appeared virtually eliminated in rats with combined H/KF or either cardiac or kidney impairment. It is interesting to note that although baseline GFR in animals with HF alone was comparable to Sham, RVP elevation induced a more marked reduction in GFR, similar to animals with KF alone or combined H/KF. This observation suggests that HF alone modulates renal hemodynamic responses to RVP elevation and may be a key instigating factor in the development of associated kidney failure. However, the virtual cessation of urine flow in all groups except the Sham control is a technical challenge which limits estimation of GFR in these experiments. As such, we were not able to detect significant differences between groups in response to RVP increase. By extension, the observation that only the control group maintained a degree of urine flow suggests that cardiac and/or renal dysfunction impairs glomerular filtration in the setting of venous congestion and that the fall in net glomerular capillary filtration pressure in these animals was greater. Given the significant blood pressure and renal hemodynamic responses to elevated RVP in all groups, these results highlight the crucial role of RVP in the cardiovascular and renal decompensation which is so problematic in the cardiorenal syndrome.

We have previously shown that an acute (Huang et al., 2018) and a chronic (Hamza et al., 2020) increase in RVP is associated with a significant reduction in blood pressure in healthy rats which is not accompanied by a reflex increase in HR—indicating modulation of the arterial baroreflex. Normally, physiological mechanisms facilitate a reflex increase in heart rate in the face of a fall in blood pressure, which would serve to optimize cardiac output to maintain organ perfusion. However, our present results similarly demonstrate a fall in blood pressure in response to RVP elevation, as well as a reduction in HR. This pattern was consistent in all groups, and the comparable ΔHR/ΔMAP is suggestive of modulation of baroreflex function similar to our earlier studies. Indeed, modulation of the arterial baroreflex such as altered sensitivity has been demonstrated in humans in response to acute venous congestion (Charkoudian et al., 2004). We (Huang et al., 2018) and others (Schmid, 1972; Kishimoto et al., 1973; Kopp et al., 1984; Bloomfield et al., 1997; Doty et al., 1999) have demonstrated that renal venous congestion is associated with activation of the renin–angiotensin system (RAS). It has been shown that ANG II sensitizes the baroreceptor reflex so that it is likely that RVP-induced suppression of HR is mediated directly by the activation of the RAS as demonstrated in our complimentary study presented in this issue (Huang et al., 2021). Collectively, our results reveal an RVP-induced impairment of blood pressure control mechanisms in rats which occurs consistently in either the healthy or pathophysiological state in which cardiac and/or renal function is compromised.

If blood pressure cannot be maintained as well in these conditions, the ensuing reduction in renal perfusion can trigger further RAS activation and renal sodium and water retention, which would not only exacerbate venous congestion and RAS activation, but may also directly contribute to progressive cardiac and renal dysfunction (Swedberg et al., 1990; Mento et al., 1996; Remuzzi et al., 2005). The fact that RVP elevation induced a fall in blood pressure in all groups, including controls is intriguing and points to the central role renal venous congestion may play in both the initiation and progression of the cardiorenal syndrome (Hinshaw et al., 1963; Damman et al., 2007; Testani et al., 2010; Uthoff et al., 2011; Ambrosy et al., 2013). In addition, it is our conjecture that the mechanism underpinning this drop in blood pressure is likely functional in nature because the fall in pressure progresses over time and is of significantly greater magnitude than any reduction in venous return imposed by the experimental partial constriction of the renal vein.

While there is no definitive mechanism yet delineated, increased RVP suppresses cardiopulmonary efferent nerve activity and ventricular contractile force, a response which is abolished by renal denervation (Kostreva et al., 1981). We and others have demonstrated that an isolated increase in RVP induces a significant suppression in renal sympathetic nerve activity (Kopp et al., 1984; Huang et al., 2018) which is considered a good reflection of systemic sympathetic outflow. However, we have also demonstrated that Ang II plays a prominent role in the hemodynamic effects imposed by acute RVP elevation in healthy animals (Huang et al., 2018). In the context of chronic renal venous congestion, it is possible that there may be an interplay between renal nerves and the RAS, particularly as the renal nerves are an important modulator of the RAS cascade. Given the profound degree of cardiovascular and hemodynamic derangement in cardiorenal syndrome, understanding the mechanism by which renal venous congestion leads to blood pressure and heart rate suppression may reveal effective treatment targets.

At first glance, one would predict the systemic and renal hemodynamic responses to RVP elevation to be more profoundly exacerbated by combined H/KF. While this might be the case in advanced cardiorenal syndrome, at the time of the acute experiment in our rodent model, baseline RVP levels were comparable between all groups. Once more pronounced venous congestion develops, we observed that the animals deteriorate rapidly and do not withstand the acute experimental procedure. In our experience, the rats with combined heart and kidney failure that had progressed to the point where venous congestion was detected did not survive beyond 20 min after induction of anesthesia (unpublished observation), which was unexpected and precluded completion of instrumentation necessary to complete the acute experiment. As such, a slightly earlier endpoint was required in order to enable this study. Although baseline RVP was comparable between all groups, multiple neurohormonal systems would be modulated to adjust to either cardiac/renal dysfunction or the combination of the two. Thus, we chose to superimpose an increase in RVP by 20 mmHg as this has been documented in the clinical literature (Mullens et al., 2009) in order to understand what the renal and hemodynamic response would be to elevated RVP before the animals were in a decompensated state. This information has value because these observed responses may reflect pathophysiological mechanisms which eventually contribute to this decompensation. Another limitation of our approach is that responses to RVP elevation were measured in anesthetized animals. This design was necessitated by the aim to simultaneously measure cardiovascular and renal parameters and the extensive instrumentation involved. Given the known effects of anesthesia on cardiovascular and neural regulation in rodents, this could account for blood pressure variability and baseline MAP values which were not significantly different, including in Sham CL + SN_x_ rats that would be expected to be significantly higher.

The most striking finding in this study is that RVP elevation induced relatively comparable responses in all animals, including controls. While this may be a reflection of the aforementioned moderate disease status in this study, it also points to an essential contribution of venous congestion and, in particular, renal venous congestion in the pathogenesis of cardiorenal syndrome. Where no dysfunction exists, as in the Sham controls, RVP elevation induced a profound depression of cardiovascular and renal control. In the case of initial cardiac or renal dysfunction alone, two conditions which feature venous congestion, it becomes apparent how an increase in RVP can not only exacerbate dysfunction in the originating organ system, but can also initiate and perpetuate dysfunction in the other. In the case of combined H/KF, volume expansion and venous congestion occur even more readily (Maxwell et al., 1950) and it is possible that elevated RVP participates in the disease state and can even play a significant role in decompensation particularly by affecting renal, but also systemic hemodynamics (Mullens et al., 2009; Iida et al., 2016; Puzzovivo et al., 2018; Xing et al., 2019; Yoshihisa et al., 2020).

### Perspectives

The fact that RVP elevation affects renal and systemic hemodynamics similarly in healthy or moderately diseased rats in our studies demonstrates the importance of either preventing or alleviating venous congestion. Strategies which aim to prevent or attenuate renal venous congestion may thus be effective therapeutic approaches in cardiorenal syndrome. Given that strategies such as diuretics and RAAS blockade are mainstays of heart failure management, it is possible that judicious use of these treatments in cardiorenal syndrome may be beneficial, provided that blood pressure is sufficiently maintained to support renal perfusion pressure. However, a comprehensive study regarding renal venous congestion—directed assessment and treatment in cardiorenal syndrome in humans is needed.

## Data Availability Statement

The original contributions presented in the study are included in the article/Supplementary Material, further inquiries can be directed to the corresponding author.

## Ethics Statement

The animal study was reviewed and approved by University of Alberta Animal Care and Use Committee (ACUC).

## Author Contributions

BB and WC conceived of and designed the experiments. SH, XH, TZ, and WZ performed the experiments. SH wrote the manuscript with the support of BB, WC, XH, TZ, and WZ. All authors participated in data analysis and interpretation, discussed the results, and approved the final manuscript.

## Funding

BB received a Grant-in-Aid from the Heart and Stroke Foundation of Canada. He also holds the Kidney Health Translational Research Chair funded by the Div. of Nephrology, Dept. Medicine, Fac. Medicine and Dentistry. XH was supported by the Li Ka Shing Foundation.

## Conflict of Interest

The authors declare that the research was conducted in the absence of any commercial or financial relationships that could be construed as a potential conflict of interest.

## Publisher’s Note

All claims expressed in this article are solely those of the authors and do not necessarily represent those of their affiliated organizations, or those of the publisher, the editors and the reviewers. Any product that may be evaluated in this article, or claim that may be made by its manufacturer, is not guaranteed or endorsed by the publisher.

## Supplementary Material

Supplementary Table S1 Baseline characterization of hemodynamic parameters. ^*^*p*<0.05, one way ANOVA, compared to Sham Control. CL, Coronary Ligation; SNx, 5/6 Subtotal Nephrectomy; MAP, Mean Arterial Pressure 594; HR, Heart Rate; EF, Ejection Fraction; RVP, Renal Venous Pressure; RVC, Renal Vascular 595 Conductance; GFR, Glomerular Filtration Rate.

Supplementary Table S2 Hematocrit at acute experiment baseline and during renal 612 venous pressure elevation.
