# Supplementary figures and images for: Chronic, Combined Cardiac and Renal Dysfunction Exacerbates Renal Venous Pressure-Induced Suppression of Renal Function in Rats
Source: Front Physiol. 2022 Feb 4;13:781504. doi: 10.3389/fphys.2022.781504 (PMC8854789; doi:10.3389/fphys.2022.781504)

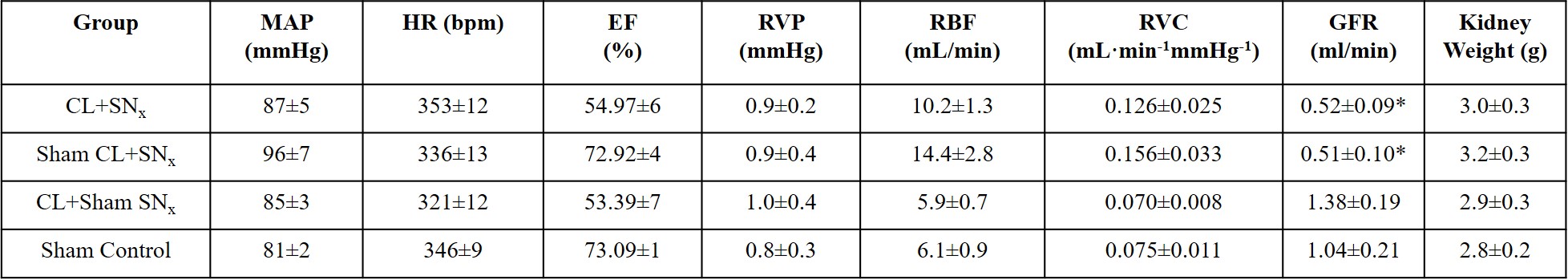

Supplement: Supplementary Table S1 — Baseline characterization of hemodynamic parameters. *p<0.05, one way ANOVA, compared to Sham Control. CL, Coronary Ligation; SNx, 5/6 Subtotal Nephrectomy; MAP, Mean Arterial Pressure 594; HR, Heart Rate; EF, Ejection Fraction; RVP, Renal Venous Pressure; RVC, Renal Vascular 595 Conductance; GFR, Glomerular Filtration Rate. [file Image_1.JPEG]

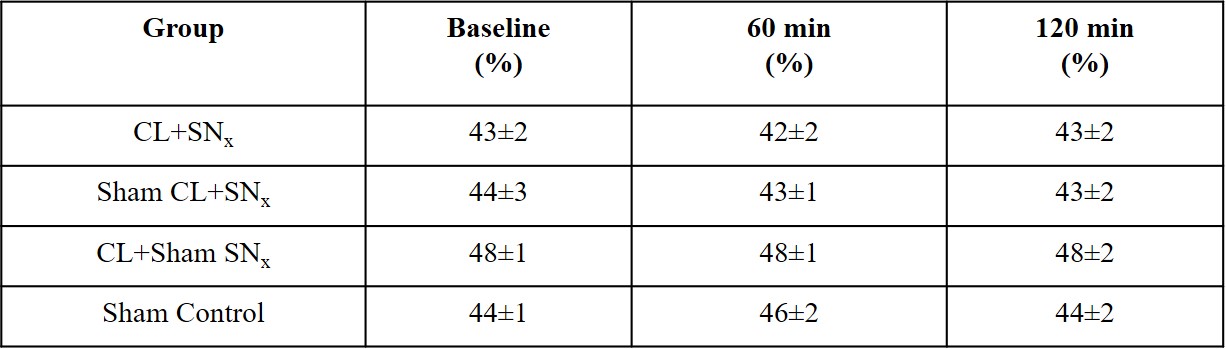

Supplement: Supplementary Table S2 — Hematocrit at acute experiment baseline and during renal 612 venous pressure elevation. [file Image_2.JPEG]
